# Local Symmetry Broken, Global Spins Aligned: Co-Emergence of Local Distortions and Antiferromagnetism in the Kagome Magnet (Fe_0.55_Co_0.45_)Sn

**DOI:** 10.1063/4.0000797

**Published:** 2025-10-27

**Authors:** Tsung-Han Yang

**Affiliations:** Oak Ridge National Laboratory, Oak Ridge, TN, USA

## Abstract

Kagome-lattice materials, such as CoSn and FeSn, have attracted significant interest as hosts of electronic flat bands and topological properties, yet their behaviors are primarily understood through average crystal structures. Here, we report the co-emergence of antiferromagnetic (AFM) order and local symmetry breaking in nominal (Fe_0.55_Co_0.45_)Sn. Using a combination of neutron powder diffraction, synchrotron x-ray total scattering, and reverse Monte Carlo modeling, we uncover simultaneous magnetic ordering and local orthorhombic distortion below T_N_ = 140 K. In this presentation, I will share comprehensive neutron and synchrotron x-ray scattering results, along with detailed analysis of short-range correlations. Our findings demonstrate that local symmetry breaking is unexpectedly coupled to global magnetic ordering, offering new insights into the interplay between structural and magnetic degrees of freedom in kagome magnets.

